# Supplementary material for: Stress among caregivers of autistic children: Conceptual analysis and verification using two qualitative datasets
Source: PLoS One. 2024 Oct 22;19(10):e0312391. doi: 10.1371/journal.pone.0312391 (PMC11495581; doi:10.1371/journal.pone.0312391)
Supplement: S1 Appendix — (DOCX) [file pone.0312391.s001.docx]

## **APPENDIX 1. Grounded theory study interview guide questions related to stress and crisis**

### Caregiver interview guide questions related to stress and crisis:

- What have been some of the possible causes of stress for you in your journey navigating care?
- When was the most urgent, stressful situation or time, or the crisis point in your journey navigating care?
- Why was it stressful or urgent? Was lack of control an issue in any way?
- How did the stress manifest itself? What consequences did it have?
- Was treatment required to address the consequences of stress?
- Is there any situation you’ve been in which you’d describe as *crisis*, defined as something that overwhelms your ability to cope or function well?

### Professional interview guide questions related to stress and crisis:

- What do you see as some of the causes and manifestations of caregiver stress in navigating interventions and information?
- Do you have any suggestions on how things could be improved reduce their stress (either generally or in your setting)?
- What would you say is the basic problem that caregivers of autistic children face with respect to information about interventions?
- Examples or descriptions of caregivers in crisis?
