# Supplementary material for: Stress among caregivers of autistic children: Conceptual analysis and verification using two qualitative datasets
Source: PLoS One. 2024 Oct 22;19(10):e0312391. doi: 10.1371/journal.pone.0312391 (PMC11495581; doi:10.1371/journal.pone.0312391)
Supplement: S2 Appendix — (DOCX) [file pone.0312391.s002.docx]

## **APPENDIX 2:** Open-ended survey questions containing stress-relevant data, number of codable responses available from each, and observations about the grounded theory categories that the coded data from each question helped replicate most

| Survey question | Codable responses | Grounded theory category(ies) that data from this question help replicate |
| --- | --- | --- |
| - To what extent do you think your physical health has been affected as a result of managing the stressors of autism in your family? You may elaborate here. | 208 | - 4) *Depleted resources reducing capacity to cope* (i.e., by sacrificing self-care)—although difficulty separating parenting stress from care navigation stress - *Consequences* on caregiver physical health |
| - To what extent do you think your mental and/or emotional health has been affected as a result of managing the stressors of autism in your family? You may elaborate here. | 217 | - 2) Buildup of multiple interacting sources - 3) Obstacles causing feelings of helplessness - *Consequences* of care navigation stress on caregiver mental health |
| - Not considering your child with autism, are there any major mental/emotional health issues in your family that add stress to your everyday life? You may elaborate here. | 210 | - 2) Buildup of multiple interacting sources—also extends this category |
| - Not considering your child with autism, are there any major physical health issues in your family that add stress to your everyday life? You may elaborate here. | 129 | - 2) Buildup of multiple interacting sources—also extends this category |
| - Have you experienced any frustrations or challenges with respect to your child(ren)’s treatment within the education system? You may elaborate here. | 95 | - 3) *Obstacles causing feelings of helplessness*, specifically within the education system |
| - Are there any other comments you would like to add to help us better understand your experiences with the impacts of services/therapies? | 132 | - 1) Sources of urgency to take action |
| - If you would like to provide any other comments about providing advocacy and support for your child, please do so here. | 70 | - 1) Sources of urgency to take action - 3) Obstacles causing feelings of helplessness |
| - If you feel they have not received optimal levels of support, can you explain the main reasons why you feel this way? Other, please explain. | 86 | - 3) Obstacles causing feelings of helplessness, outside the education system |
| - If you have any feedback or anything else you want to add, please do so here. | 79 | - Various categories |
