# Supplementary material for: Stress among caregivers of autistic children: Conceptual analysis and verification using two qualitative datasets
Source: PLoS One. 2024 Oct 22;19(10):e0312391. doi: 10.1371/journal.pone.0312391 (PMC11495581; doi:10.1371/journal.pone.0312391)
Supplement: S3 Appendix — (DOCX) [file pone.0312391.s003.docx]

## **APPENDIX 3:** Summary of stress-related coding of survey data, organized under the four major grounded theory study-derived categories of stress

z_: New code (not part of original grounded theory study coding structure

References: Number of examples (survey data segments) coded to that code

| Code Name | References | | Description |
| --- | --- | --- | --- |
| 1_Sources urgency to take action | 2 | Included feelings of anxiety about possible future outcomes of autism (including when coming to understand the child has autism, and encountering significant obstacles to intervention), seeing one’s child struggle, knowing the importance of intervening early, learning it’s up to caregivers to take action, and guilt for not doing enough. | |
| (Guilt for) not doing enough | 7 |  | |
| Anxiety re future | 31 | feelings of anxiety about possible future outcomes of autism (including when coming to understand the child has autism, and encountering significant obstacles to intervention) | |
| Over 18 | 4 |  | |
| Knowing early intervention important | 10 | knowing the importance of intervening early | |
| Seeing child struggle | 32 | seeing one’s child struggle | |
| Up to caregivers | 10 | learning it’s up to caregivers to take action | |
| 2_Multiple Interacting Sources | 0 | (ie, non-navigation-related sources) Stress attributable to the overwhelming demands of navigating intervention sometimes coincided in time with things like stress due to problematic child behaviors, demands of another newborn, needs of typical siblings, death in the family, or physical aspects of caregivers’ daily living environment. | |
| Child behaviors | 17 |  | |
| Death_Loss in the family | 10 | Includes serious health impacts (eg, stroke) on family (grandparents). | |
| z_Dependent (grand)parents relatives | 22 |  | |
| Demands of multiple children | 86 |  | |
| Another newborn | 1 |  | |
| Needs of typical siblings | 9 |  | |
| z_Multiple Autistic children | 19 |  | |
| z_Other children with high needs | 56 |  | |
| Physical living environment | 2 |  | |
| Transportation limitations | 1 |  | |
| Employment demands | 9 |  | |
| Lost job_uncertainty | 7 |  | |
| Marital (separation) issues | 12 | Includes divorced or separated parents, and their disputes about sharing parenting or the costs. | |
| Caregiver prior mental health | 39 |  | |
| z_Caregiver prior physical conditions | 56 | Includes medical conditions. | |
| z_Caregiver psychiatric (non-anxiety or mood disorders) | 17 |  | |
| z_Caregiver has autism | 8 |  | |
| z_Spouse mental health_supporting | 2 |  | |
| 3_Obstacles causing feeling helplessness | 1 | Obstacles causing feelings of helplessness: Often experienced as setbacks to obtaining intervention, with potential to cause a sense of helplessness (loss of control), despair (emotional loss), and reduced capacity to cope emotionally. | |
| Effects | 1 |  | |
| Despair_Emotional loss | 14 | Includes sense of loss of hope. | |
| Helplessness_Loss of control | 22 |  | |
| Reduced coping capacity | 13 |  | |
| z_Preventing a better life | 2 | ...for the caregiver or family | |
| Seen as unnecessary | 1 | Obstacles perceived as unnecessary. | |
| Setbacks to accessing | 2 | Setbacks to obtaining intervention | |
| Complexity_Not knowing enough | 16 | Not knowing how to navigate because it is too complex | |
| Alone_Needing support to navigate | 17 |  | |
| Misinformation_missed opportunities | 2 |  | |
| Cut off from service | 5 |  | |
| Not qualifying | 8 | Or uncertainty about qualifying for funded intervention | |
| Transportation burden | 11 |  | |
| Unavailable services | 16 |  | |
| Beyond 18 | 10 |  | |
| Geographic lack of availability | 6 | Service(s) not available locally | |
| Mental health services for child | 4 | Mental health services for child unavailable | |
| Social activities | 4 | Including in school | |
| Waitlists_Booked | 40 |  | |
| Diagnosis | 3 | Obstacles to diagnosis | |
| Education | 6 |  | |
| ABA_therapist access limited | 7 |  | |
| Bureaucratic_time wasting | 9 |  | |
| Communication poor | 3 |  | |
| Disagreements_Conflict | 24 |  | |
| Refusing to accommodate | 10 |  | |
| Expertise lacking | 20 |  | |
| Inflexibility | 7 | of school, principal, other educators | |
| Caregiver expertise not respected | 7 |  | |
| Planning-IEP not followed | 7 |  | |
| Policy violation | 12 |  | |
| Human rights violation | 6 |  | |
| Punitive | 4 |  | |
| Remove-switch from school | 14 | Pull from school, sometimes to do homeschool instead, sometimes to switch schools. | |
| School lacks capacity to keep child in school | 31 | Not equipped to deal with child, and keep them in school | |
| Child unsafe at school | 12 |  | |
| Restraining child_Police called | 6 |  | |
| Financial constraint_High cost | 8 | Financial constraint, meaning having inadequate funds that specifically prevents accessing desired intervention. It is a cause of stress because it is a barrier, not because it causes financial uncertainty for other aspects of life. | |
| Having to fight | 18 |  | |
| Hours conflict w work | 7 | Services are only available at hours when the caregiver cannot come, or only come at great sacrifice | |
| Not being heard | 1 |  | |
| Restricted choice_variety_ABA only | 7 |  | |
| 4_Depleted resources_Exhaustion | 5 | Depleted resources reducing capacity to cope: Progressing deficits in expendable resources (time, financial, physiological reserves for coping physically, emotionally, and cognitively) to meet the work-related demands of autism (parenting and navigating intervention) were a form of stress analogous to exhaustion because they compromised caregivers’ capacity to handle further challenges. Caregivers often responded by shutting down further resource use, or seeking balance. Includes reference to exhaustion. | |
| 1_Time burden | 82 |  | |
| Employment time conflict | 21 | Time off work (includes that due to removing child from school) | |
| Retrieve child from school | 9 | Being called to pick up child and take home from school because of behavioural problems. Requires the caregiver to interrupt day, and spend rest of day caring for child. | |
| Self health care | 5 |  | |
| Eating Healthy food (preparation) | 5 |  | |
| Exercising less | 1 |  | |
| z_Seeing personal doctor | 5 |  | |
| z_Single parenting | 4 |  | |
| Other parent working | 2 |  | |
| Sleep loss | 6 |  | |
| 2_Financial burden | 42 |  | |
| 3_Physical burden | 12 |  | |
| 4_Emotional burden | 39 |  | |
| 5_Cognitive energy burden | 8 |  | |
| Needing break_Exhaustion | 23 |  | |
| Restoring balance | 1 |  | |
| Consequences | 0 |  | |
| Anxiety | 22 |  | |
| PTSD | 3 | any reference to this term | |
| Depression | 22 |  | |
| Divorce | 1 |  | |
| Family dysfunction | 11 | (anger, blame, conflict) | |
| Financial distress | 4 |  | |
| Guilt | 10 | Often, guilt for not doing enough. Therefore it also relates to "Depleted resources reducing capacity" | |
| z_Having to prove need | 1 |  | |
| Marital dysfunction | 9 | (anger, blame, conflict) | |
| Neglect family members | 4 |  | |
| Obsessiveness | 0 |  | |
| Overwhelmed, Becoming | 11 | Possibly: (manifesting as denial, avoidance). | |
| Self-neglect | 15 | (disrupted sleep, eating, self-care) | |
| Social consequences (of stress) | 5 |  | |
| z_Employment | 2 | Threats to job security or loss or discontinuation of a job | |
| Physical health | 5 |  | |
| Fitness lost | 1 |  | |
| Sleep lost | 8 | May be related to anxiety, or lost time (i.e., broad). | |
| Weight gain | 3 |  | |
| Crisis | 1 | An event occurring when stress (due to parenting, navigating, etc.) reaches or passes a threshold level that disrupts homeostasis of the affected caregiver-related functioning system (psychological self, physical self, family, or relationship), resulting in a sudden or progressive loss of function, and sometimes the sense that a catastrophic failure is imminent. Crisis compels the caregiver to respond, seeking resolution or change from the status quo to restore balance; rarely, failure of the system occurs. | |
| Child | 5 | As a functioning system that can go into crisis. | |
| Family | 9 | As a functioning system that can go into crisis. | |
| Marital unit | 4 | As a functioning system that can go into crisis. | |
| Caregiver-child unit | 1 | As a functioning system that can go into crisis. | |
| Self_Emotional-Psychological | 14 | As a functioning system that can go into crisis. | |
| Self_Financial | 4 | As a functioning system that can go into crisis. | |
| Self_Physical | 3 | As a functioning system that can go into crisis. | |
