# Supplementary material for: Stress among caregivers of autistic children: Conceptual analysis and verification using two qualitative datasets
Source: PLoS One. 2024 Oct 22;19(10):e0312391. doi: 10.1371/journal.pone.0312391 (PMC11495581; doi:10.1371/journal.pone.0312391)
Supplement: Attachment — Submitted filename: Response to Reviewers_2024-07-24.docx [file pone.0312391.s004.docx]

**Response to Reviewers Letter**

PONE-D-24-18422

Stress among caregivers of autistic children: Conceptual analysis and verification using two qualitative datasets

PLOS ONE

2024-Jul-05

EDITOR COMMENTS:

1. Please ensure that your manuscript meets PLOS ONE's style requirements, including those for file naming. The PLOS ONE style templates can be found at <https://journals.plos.org/plosone/s/file?id=wjVg/PLOSOne_formatting_sample_main_body.pdf> and <https://journals.plos.org/plosone/s/file?id=ba62/PLOSOne_formatting_sample_title_authors_affiliations.pdf>.

AUTHOR RESPONSE:

We have taken care to follow style requirements as outlined in the templates in the initial submission. Please advise if further changes needed.

2. We note that the grant information you provided in the ‘Funding Information’ and ‘Financial Disclosure’ sections do not match.

When you resubmit, please ensure that you provide the correct grant numbers for the awards you received for your study in the ‘Funding Information’ section.

AUTHOR RESPONSE:

Of the multiple funding sources described in the Financial Disclosure, only one has a grant number. We have added this number to the following revised Financial Disclosure statement, and replaced names with initials:

“SJG received a grant from Autism Ontario to support development of this manuscript (grant number N/A). SJG completed the survey data analysis during a postdoctoral fellowship at Wilfrid Laurier University, funded by a gift to JM and the Laurier Autism Research Consortium from the Dare Family, in honor of the late Carl M. Dare (grant number N/A). SJG completed the primary grounded theory study during his doctorate at McMaster University, with the support of two Strategic Training Initiative in Health Research fellowships funded by the Canadian Institutes of Health Research—an Autism Research Training Fellowship jointly funded by the Sinneave Family Foundation, and a Knowledge Translation Canada Fellowship (grant: CIHR 2777601301). JM received an internal grant from Wilfrid Laurier University to support the survey (grant number N/A). The funders had no role in study design, data collection and analysis, decision to publish, or preparation of the manuscript.”

We have added the additional missing funding sources to the Funding Information section.

3. We note that you have indicated that there are restrictions to data sharing for this study. PLOS only allows data to be available upon request if there are legal or ethical restrictions on sharing data publicly. For more information on unacceptable data access restrictions, please see <http://journals.plos.org/plosone/s/data-availability#loc-unacceptable-data-access-restrictions>.

Before we proceed with your manuscript, please address the following prompts:

a) If there are ethical or legal restrictions on sharing a de-identified data set, please explain them in detail (e.g., data contain potentially identifying or sensitive patient information, data are owned by a third-party organization, etc.) and who has imposed them (e.g., a Research Ethics Committee or Institutional Review Board, etc.). Please also provide contact information for a data access committee, ethics committee, or other institutional body to which data requests may be sent.

b) If there are no restrictions, please upload the minimal anonymized data set necessary to replicate your study findings to a stable, public repository and provide us with the relevant URLs, DOIs, or accession numbers. For a list of recommended repositories, please see
<https://journals.plos.org/plosone/s/recommended-repositories>. You also have the option of uploading the data as Supporting Information files, but we would recommend depositing data directly to a data repository if possible.

We will update your Data Availability statement on your behalf to reflect the information you provide.

AUTHOR RESPONSE:

We have updated our statement on data sharing as below.

From (OLD statement): Data cannot be shared publicly because of ethical privacy and confidentiality requirements related to the qualitative interview data.

To (NEW statement): Data cannot be shared publicly because of ethical privacy and confidentiality requirements related to the grounded theory study’s qualitative interview data (as enforced by the Hamilton Integrated Research Ethics Board (HiREB)), and the survey study’s qualitative survey data (as enforced by the Wilfrid Laurier University Research Ethics Board). The enforcement by both REBs is governed by Canada’s Tri-Council Policy Statement: Ethical Conduct for Research Involving Humans – TCPS 2 (<https://ethics.gc.ca/eng/tcps2-eptc2_2022_chapter5-chapitre5.html#2>), and corresponds to the required legal and ethical assurances provided to participants in the consent process about how their privacy and the confidentiality of their data would be protected. Grounded theory or survey data are available for researchers who meet the criteria for access to such confidential data after first contacting either the HiREB ([fspence@mcmaster.ca](mailto:fspence@mcmaster.ca)) or Wilfrid Laurier University REB ([REB@wlu.ca](mailto:reb@wlu.ca)), respectively.

4. Please include your full ethics statement in the ‘Methods’ section of your manuscript file. In your statement, please include the full name of the IRB or ethics committee who approved or waived your study, as well as whether or not you obtained informed written or verbal consent. If consent was waived for your study, please include this information in your statement as well.

AUTHOR RESPONSE: This information was in the Methods section of the first submission of the manuscript, but it was embedded in the respective descriptions of the two component studies, and not in a separate “Ethics statement”. We have now moved this information under a new “Ethics statement” subhead at the end of the Methods section.

5. Please review your reference list to ensure that it is complete and correct. If you have cited papers that have been retracted, please include the rationale for doing so in the manuscript text, or remove these references and replace them with relevant current references. Any changes to the reference list should be mentioned in the rebuttal letter that accompanies your revised manuscript. If you need to cite a retracted article, indicate the article’s retracted status in the References list and also include a citation and full reference for the retraction notice.

AUTHOR RESPONSE: To the best of our knowledge the reference list is complete and correct and does not include any retracted papers.

REVIEWER #1 COMMENTS:

No comments requesting changes.

REVIEWER #2 COMMENTS:

1. Introduction: Lines 92-94 seem to repeat lines 69-72.

AUTHOR RESPONSE: While the two sentences mentioned reference a similar concept (*expendable resource loss*), we respectfully suggest they make separate points that are needed to provide important background in the Introduction, and are not redundant. The earlier sentence (lines 69-72), “Combined, these tasks require considerable work, rapid development of expertise (knowledge, skills), and expendable personal resources (time, energy and financial resources) from the caregiver,” describes the burden of the tasks of navigating intervention including the loss of expendable resources. The purpose of the later sentence (lines 92-94), “In addition to being a primary cause of stress, however, care navigation has potentially measurable impacts on personal and family time, energy, and financial resources,” is to highlight how downstream impacts of care navigation (i.e, the three types of *expendable resource loss*) represent potentially measurable aspects of caregiver stress attributable to care navigation.

2. Methods: Was the survey data of sufficient depth? Potential bias in terms of some caregivers providing a lot of detail and others giving one word answers, therefore only some responses can be used for qualitative analysis?

AUTHOR RESPONSE: Indeed, as we describe in the methods, there was variation in the level of data provided in response to the survey questions that we chose to analyze as containing potentially relevant data (lines 209-215): “Unlike questions in the grounded theory interview guide, these asked less directly about stress or crisis, and several questions addressed service-related aspects expected to be associated with stress. Codable responses ranged from several words to several lines of participant-typed text.” We feel, however, that we have acknowledged and discussed the limitations of this data as a key topic in the Discussion: “While survey data can be useful to extend conceptual analyses, we note two limitations of this data type. First, open participant-typed responses were noticeably less articulate than for transcribed interviews—due to typos, abbreviations, inattentive writing, varying literacy levels, etc. Unlike interviews, surveys do not allow returning to clarify intended meanings. Second, due to their brevity and typical focus on single timepoints, open survey questions are poorly suited to gathering information needed to understand action as a process that evolves over time or about relative timing of events needed to understand unfolding narratives. Reflecting this limitation, one respondent articulated frustration with not being able to share more within the survey format about how certain stressors had changed over the course of her journey. Grounded theory interviewing, by contrast, is suited to reflect process and narrative timelines [36, 37]—in this case, the evolving nature of and caregiver responses to stress over their trajectory navigating autism-related care.” We respectfully suggest that this sufficiently describes the types of “bias” (limitations) embodied in the survey data, specifically with respect to the analytic objectives, and that no additional description is necessary.

3. The methods might benefit from some additional sub-headings

AUTHOR RESPONSE: We have followed this advice by adding the following sub-headings: We regrouped content from both studies related to ethics under a new subheading “Ethics statement.” Additionally, we reworded the two second-level headings to reference the study data being used (to “Primary conceptual analysis of grounded theory data,” and “Conceptual verification and extension using survey data”), and added third-level headings under each.

4. Results: Lines 309-318: Some of the findings seem to be caregivers discussing another caregiver – was the study aiming to capture third-hand data such as this and can it be evaluated in the same way as a participant discussing their own experience?

AUTHOR RESPONSE: In the study, we accepted secondary reports (i.e., other informants’ observations of parents) of caregivers navigating care as key data in this study, both from the 9 professionals interviewed and from parent participants. Such data were considered analytically useful if they could be used in the development of a category or concept. In the case of the lines cited, this data served (i.e., provided a case example) to develop the concept of crisis attributable to a single event (i.e., a medical emergency). This is consistent with grounded theory methods, where it is acceptable to turn to different sources for data that help in conceptual development (e.g., document data, literature review, observation, and different informants).

5. If ethics allows, it would be useful to include some information with quotes, such as the child’s age and severity of autism to identify whether the quotes represent a range from the sample.

AUTHOR RESPONSE: Thank you for this suggestion. We have added information to accompany quotes that describes the parent participant in terms of child age, autism severity, siblings, marital status in some cases, and urban-rural status in some cases. We note that all quotes are from unique participants except one parent who provided two quotes (line 300, and final quote in Table 1).
